# Different Faces of Rathke’s Cleft Cyst

**DOI:** 10.5334/jbsr.3047

**Published:** 2023-03-03

**Authors:** Clara Brou, Idil Gunes Tatar

**Affiliations:** 1 Cliniques universitaires Saint-Luc, BE

**Keywords:** Rathke’s cleft cyst, MRI, cystic sellar lesion

## Abstract

Rathke’s cleft cysts (RCC) are rare benign cystic lesions that might present with different imaging features, potentially posing a problem in the radiological diagnosis of cystic sellar lesions. The purpose of this pictorial review is to present an overview of the radioclinical features of RCC through four clinical cases with different radiologic findings confirmed by pathology, as well as reviewing the common differential diagnosis to be considered.

The subjects are women, aged 11 to 73 who underwent recent transsphenoidal surgical resection with a postoperative follow-up period of a few months to three years.

## Introduction

Rathke’s cleft cysts (RCCs) are benign sellar lesions arising from epithelial remnants of Rathke’s pouch that originate between the anterior and posterior lobes of the pituitary gland with a peak in third to fifth decades of life [1]. The residual lumen of the pouch is reduced to a narrow Rathke’s cleft, which generally regresses, and the persistence and enlargement of this cleft is believed to be the origin of an RCC [2].

Most RCCs are small and asymptomatic, but larger cysts may compress adjacent neurovascular structures and symptoms may arise, such as headache, endocrine dysfunction, and occasionally visual disturbances.

Diagnosis of RCC is strongly suggested at magnetic resonance imaging (MRI). Typical imaging findings include a nonenhancing, noncalcified cyst located between the anterior and posterior lobes of the pituitary gland with sharp contours as well as close contact with the posterior lobe and strict midline location [3]. RCCs may show various signal intensities on T1- and T2-weighted images, depending on its cystic content.

Routine clinical follow-up with MRI is sufficient for incidental asymptomatic cysts. For rare cases with symptomatic lesions, treatment most often consists of surgery, usually an endonasal transsphenoidal approach.

Although considered to be benign lesions, RCCs commonly recur. The recurrence rate may depend on the size of the cyst, presence of squamous metaplasia, or partial excision of the paper-thin wall of the cyst, leaving in place the secretions that may re-accumulate or even result in intraoperative cerebral spinal fluid leak and leading to need for an abdominal fat graft or sellar packing [1].

## Presentation of the Cases

### Classical Cystic RCC

**Figure 1:**
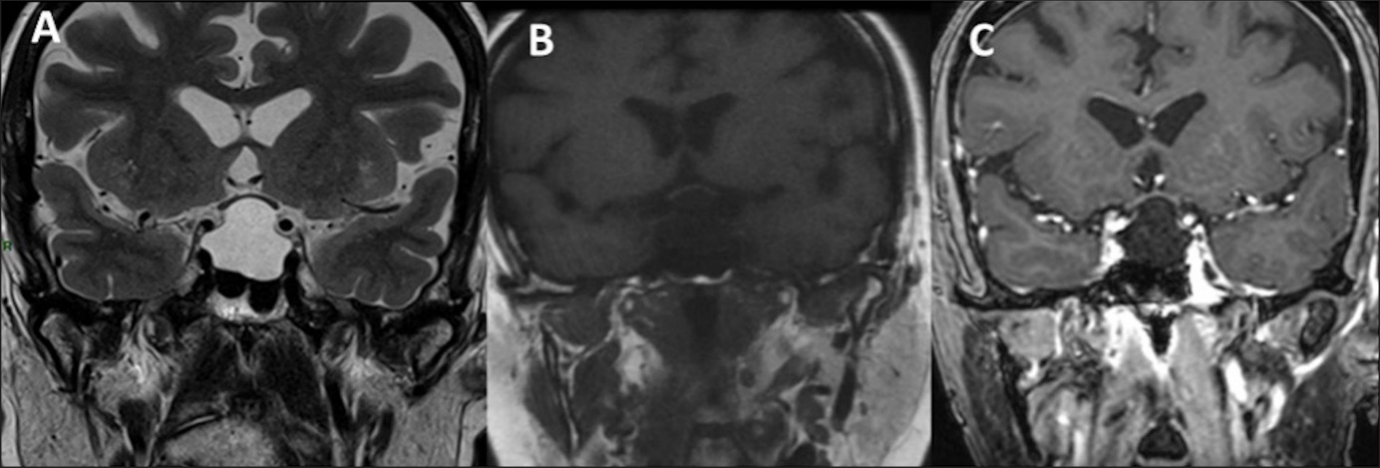
Intrasellar and suprasellar lesion in a 67-year-old female patient, which is hyperintense in on coronal T2WI **(A)**, hypointense on coronal T1WI **(B)**, and no enhancement is observed on contrast-enhanced T1WI **(C)** diagnosed as Rathke’s cleft cyst pathologically.

A 67-year-old woman presenting with tiredness and weight gain was diagnosed with a pituitary insufficiency. MRI evaluation revealed a large cystic intrasellar and suprasellar lesion with superior displacement of the optic chiasm, posterior displacement of the neurohypophysis, and extension to the cavernous sinuses (Figure 1).

**Figure 2:**
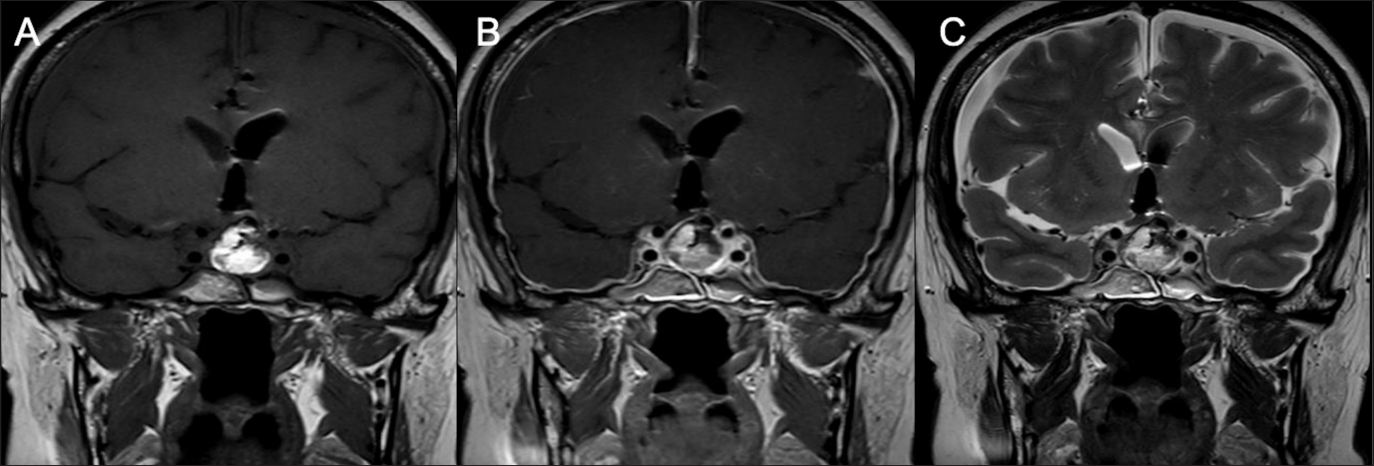
MRI after first surgery: Coronal T1WI **(A)**, Coronal post-contrast T1WI **(B)** and coronal T2WI revealed signs of intracranial hypotension: right and left subdural collections and pachymeningeal thickening and enhancement.

Patient underwent transsphenoidal surgical resection, which confirmed the diagnosis of RCC. Immediate post-operative follow-up revealed a sphenoidal breach with CSF flow leading to intracranial hypotension which was followed up by MRI (Figure 2) and a surgical revision a few months later to clog it.

The postoperative follow-up revealed headaches that were not present before her first operation, persistent pituitary insufficiency requiring hormone substitution therapy: gonadotropic (LH FSH abnormally low for menopause), thyrotropic (low T4 and low TSH), somatotropic (collapsed GH), and corticotropic (low cortisol and ACTH), and slightly elevated but stable prolactin probably related to pituitary stem lesion. No other problems were reported, including no recurrence so far.

### RCC with High Protein Content

**Figure 3:**
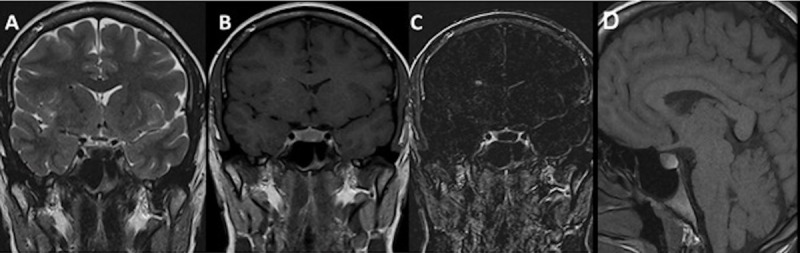
Intrasellar lesion in a 22-year-old female patient with a hypointense component on coronal T2WI **(A)**, which is hyperintense on coronal **(B)** and sagittal **(D)** T1WI, and without enhancement on coronal contrast-enhanced substracted T1WI **(C)** diagnosed as Rathke’s cleft cyst pathologically.

A 22-year-old woman who presented with chronic headaches underwent an MRI examination, which showed a cystic intrasellar lesion with high protein content. No mass affect was observed (Figure 3). Patient underwent transsphenoidal surgical resection with complete resection of the RCC and resolution of headaches in her follow-up. No post-operative complication and no recurrence so far were reported.

### RCC with a Mural Nodule

A 73-year-old woman presented with an incidental cystic pituitary lesion discovered in context of malaise with loss of consciousness. The hormonal assessment during hospitalization revealed a pituitary insufficiency affecting the gonadotropic, thyrotropic, corticotropic, and somatotropic functions as well as hyperprolactinemia compatible with a compression of the pituitary stem.

**Figure 4:**
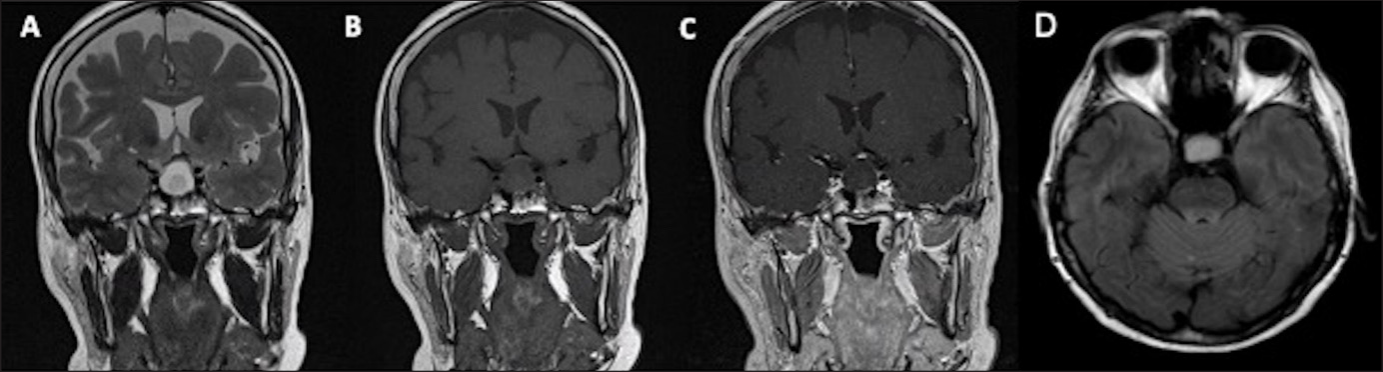
Coronal T2WI **(A)**, coronal T1WI **(B)**, coronal contrast-enhanced T1WI **(C)** and axial FLAIR **(D)** images in a 73-year-old female patient show cystic intrasellar and suprasellar lesion with a non-enhancing intracystic nodule hyperintense to surrounding fluid on T1 and hypointense on T2.

MRI examination demonstrated a cystic sellar and suprasellar lesion with a non-enhanced intracystic nodule (Figure 4). An intracystic nodule can be identified, which is virtually pathognomonic of a RCC with an incidence of 37.5–45% [4] and can show various signal intensities depending on the signal of the surrounding fluid visibility.

**Figure 5:**
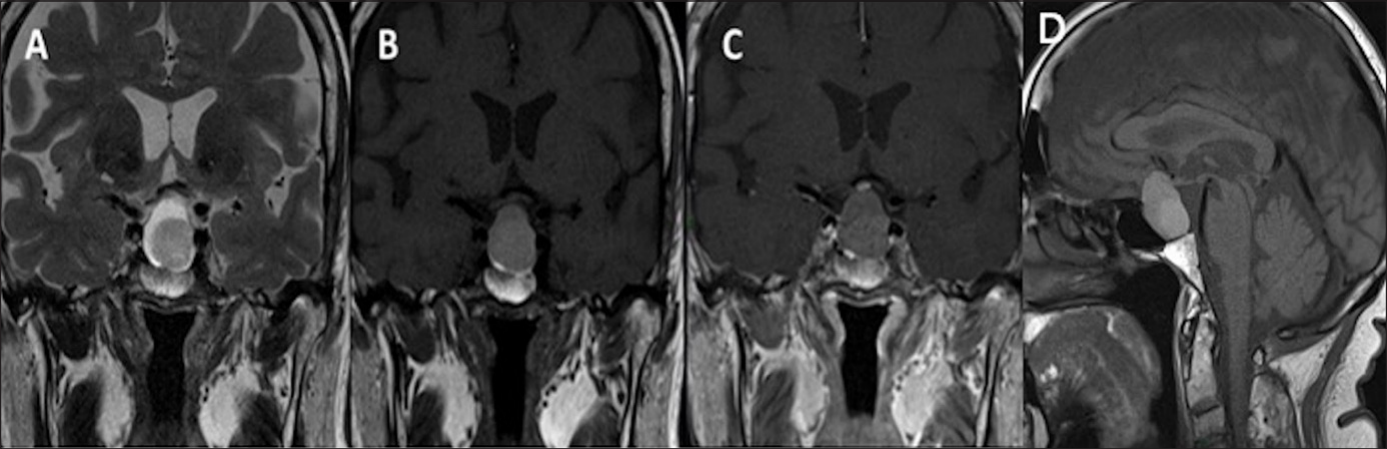
Ten-year follow-up of the same patient: Coronal T2WI **(A)**, T1WI **(B)**, post-contrast T1WI **(C)** and sagittal T1WI **(D)** show an increase in size of the lesion as well as the intracystic nodule.

In the course of her follow-up, a significant radiological progression of the cyst was observed at the end of the ten-year follow up. MRI revealed an increase in the size of both the cystic lesion and the intracystic nodule showing chiasmatic mass affect and without extension to the cavernous sinuses (Figure 5).

Considering the radiological progression of the cyst and given the context, the patient underwent surgical resection after discussion at a multidisciplinary oncology meeting. In the postoperative course, persistence of a relatively important pituitary insufficiency was revealed, justifying the continuation of hormone substitution therapy. No post-operative complication or recurrence has been observed so far.

### RCC with Heterogeneous Texture and Peripheral Enhancement

**Figure 6:**
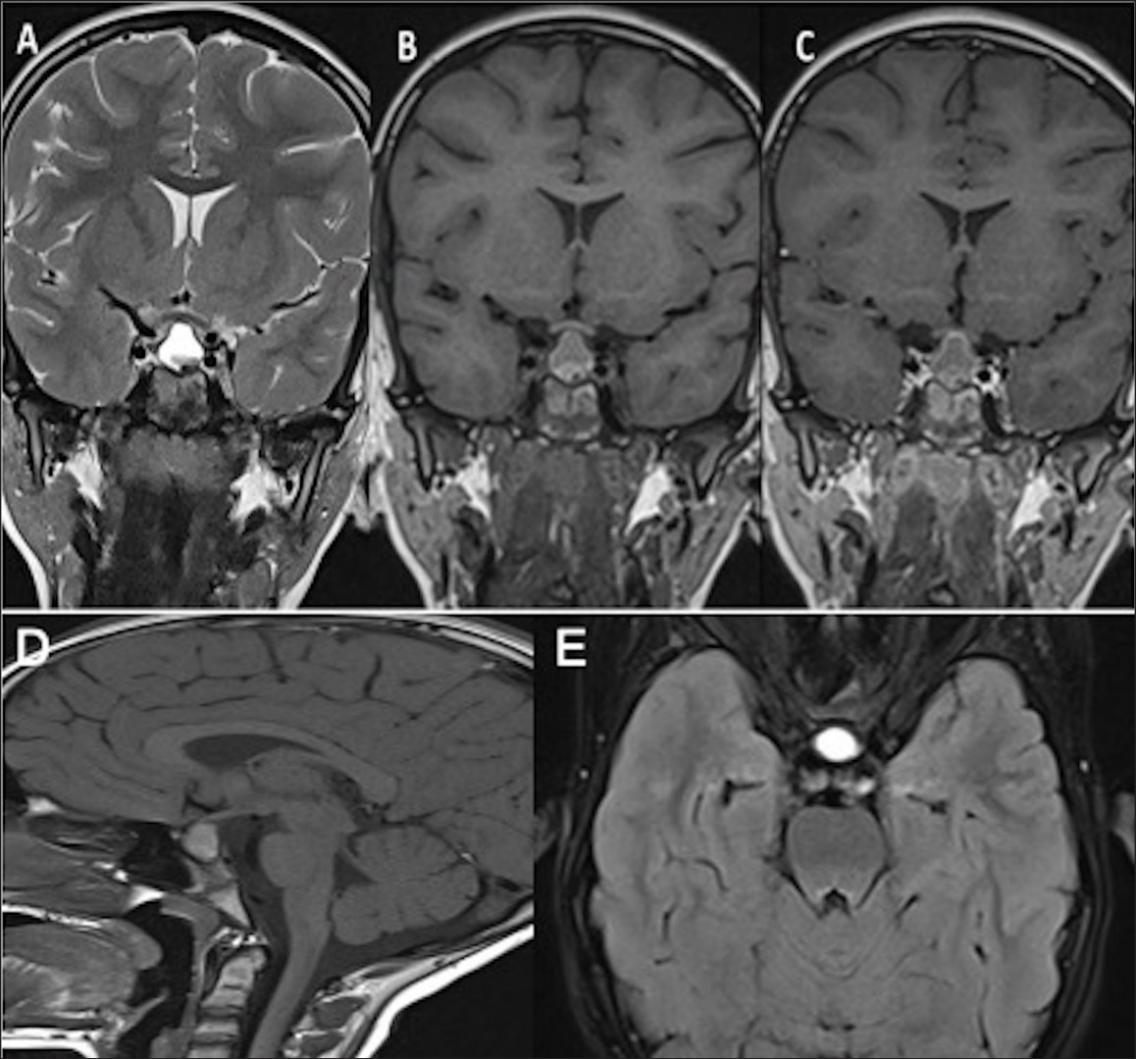
Coronal T2WI **(A)**, T1WI **(B)**, post-contrast T1WI **(C)**, sagittal T1WI **(D)** and axial FLAIR **(E)** images demonstrate a sellar and suprasellar lesion with heterogeneous content in the lower part of the lesion and peripheric enhancement.

A 11-year-old female patient who presented with failure to thrive and diabetes insipidus underwent an MRI examination, which revealed a cystic intrasellar and suprasellar lesion with heterogeneous content with an irregular solid component and peripheral enhancement coming into contact with the optic chiasm (Figure 6).

Patient underwent transsphenoidal surgical resection without immediate post-operative complication, and no recurrence has been observed so far. During her postoperative follow-up, a nice recovery of her growth curve was reported. Initially, a decrease in her growth hormone level was reported, for which a substitution treatment was continued for a few months in addition to the antidiuretic hormone therapy.

## Discussion

Although the recurrence rate of RCCs appears to be relatively high, none of our patients have demonstrated postoperative recurrence to date. This can be explained probably by their short follow-up period after surgery, ranging from a few months to three years, which can be considered a limitation in our case series.

There is currently no clinical consensus on the best strategy to treat recurrences, but radiotherapy seems to be a good emerging alternative to surgery in RCC treatment, according to Chalif, especially for the cases that tend to recur [5].

In our study, all patients underwent transsphenoidal surgical resection, but it should be kept in mind that surgery exposes the patient to a risk of postoperative complications and RCC treatment remains controversial for some authors. Based on Truong’s study, surgery would appear to be particularly suitable for treating visual disturbances, and regular follow-up is more appropriate than surgery regarding headaches, hyperprolactinemia, endocrine disruptions, and diabetes insipidus [6]. This was confirmed in our clinical cases, except for case 2, where the surgery demonstrated a beneficial effect on the patient’s headaches.

Diagnosis of RCCs might be challenging because they can mimic other intra- or suprasellar lesions, such as cystic pituitary adenoma and cystic craniopharyngioma, or less frequently, arachnoid cyst, epidermoid cyst, mucocele, abscess, or pituitary apoplexy [7]. RCC is sometimes indistinguishable from cystic pituitary adenoma on MRI. In the literature the presence of a fluid-fluid level (presumably related to intracystic hemorrhage), septation, an off-midline location, suprasellar extension with typically a ‘figure of eight’ appearance and the absence of an intracystic nodule are important imaging features in favor of a pituitary adenoma [4,8].

On the other hand, suprasellar localization, the presence of calcification or a solid enhancing component is characteristic for craniopharangioma [3]. Histologically craniopharyngiomas present as two types: squamous-papillary and adamantinous. The first type is mostly a solid suprasellar tumor in adults with the presence of intensely enhancing and small necrotic areas on MRI. The second type is a predominantly cystic tumor with peripheral calcifications, found in children [8].

Cystic portions of both pituitary adenoma and craniopharyngioma can show an enhancing thick wall which can be attributed to squamous metaplasia, inflammation, deposition of haemosiderin, or cholesterol crystals in the cyst wall [8].

## Conclusion

We describe a wide spectrum of radiological features that are dependent on cystic content and that a radiologist should be aware of to make a correct diagnosis. Although radiological findings can be nonspecific, some MRI features may be helpful in differentiating it from other well-circumscribed intrasellar processes; nonetheless sometimes surgery is necessary to lead to the definitive diagnosis.

It is also important to point out that even though RCCs are benign changes pathologically every RCC case should have serious and rigorous multidisciplinary follow-up, primarily because of its location regarding compression effects and multiple important pituitary functions. Indeed, according to the literature, one RCC case reported by Sawano resulted in cardiac arrest induced by a lack of cortisol [9].
